# Floating ascending aortic thrombus with antiphospholipid syndrome: a case report

**DOI:** 10.1186/s44215-024-00174-2

**Published:** 2024-11-08

**Authors:** Shinya Tajima, Tomoaki Kudo, Daisuke Mori, Katsukiyo Kitabayashi

**Affiliations:** Department of Cardiovascular Surgery, Kansai Rosai Hospital, 3-1-69, Inabaso, Amagasaki, Hyogo 660-8511 Japan

**Keywords:** Ascending aortic thrombus, Antiphospholipid syndrome, Computed tomography angiography

## Abstract

**Background:**

Ascending aortic thrombus is a rare disease that can cause fatal thromboembolism. The treatment for the disease is not well defined and depends on the clinical experience of surgeons. Most reports of thrombosis in antiphospholipid syndrome (APS) are associated with venous or peripheral arterial thrombosis, and there are almost no reports of thrombosis of the aorta.

**Case presentation:**

A 74-year-old male was referred to our department with claudication of the left leg lasting 3 months. A computed tomography angiography (CTA) and a transthoracic echocardiography demonstrated that a floating and pedunculated mass associated with APS was located at ascending aortic lumen and an embolism in the left superficial femoral artery. Under deep hypothermic circulatory arrest, we resected a floating mass without the graft replacement. CTA 1 year after surgery showed no recurrence of thrombus.

**Conclusion:**

We experienced a rare case of floating ascending aortic thrombi. As in this case, we consider that a floating ascending aortic thrombus with embolic events should be performed by surgical intervention.

## Background

Aortic floating thrombus is recognized in approximately 9% of patients presenting with arterial thromboembolism [1]. In particular, floating thrombus in the ascending aorta is rare and fatal. Treatment of this disease is not well defined and largely depends on the clinical experience of the surgeon. We present a rare case of an ascending aortic floating thrombus associated with antiphospholipid syndrome (APS) and discuss its management.

## Case presentation

**Fig. 1:**
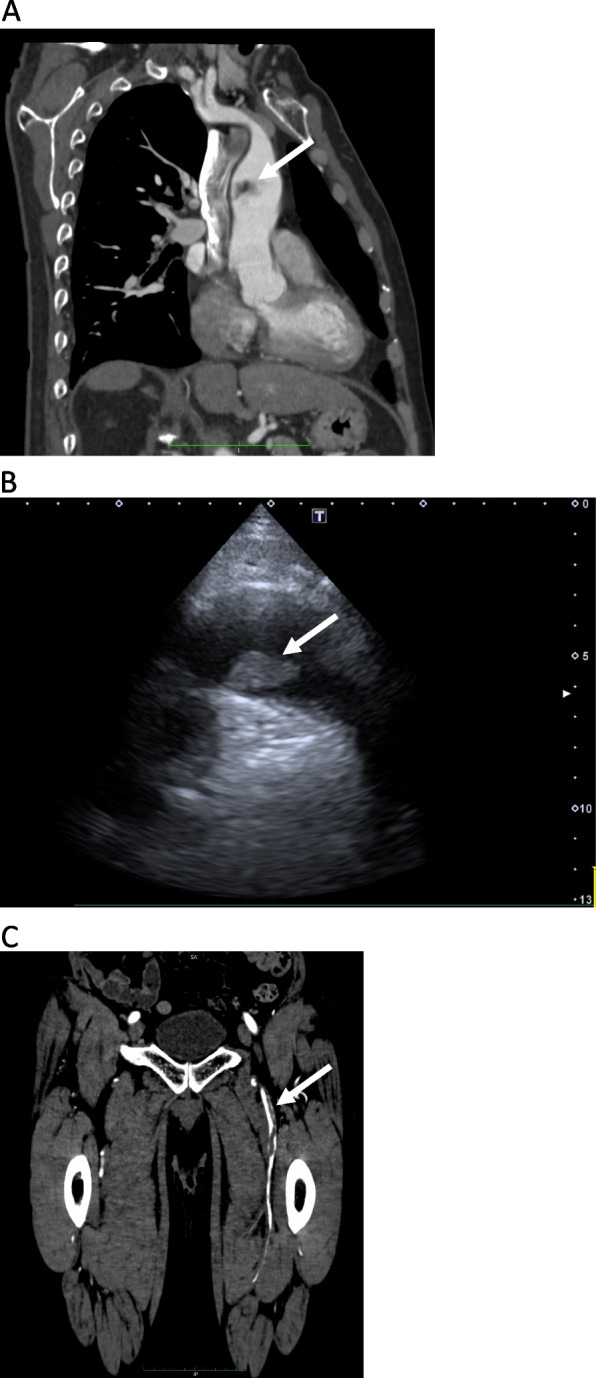
A computed tomography angiography (**A**) and a transthoracic echocardiography (**B**) demonstrated that a floating mass measuring 23 × 14 mm and 26 × 13 mm was located at ascending aortic lumen and an embolism in the left superficial femoral artery (**C**)

A 74-year-old male was referred to our department with claudication of the left leg lasting 3 months. He had hypertension, dyslipidemia, and bladder cancer which was complete remission and no adjuvant chemotherapy. A computed tomography angiography (CTA) and a transthoracic echocardiography demonstrated that a floating and pedunculated mass was located at ascending aortic lumen (Fig. 1A and B) and an embolism in the left superficial femoral artery (Fig. 1C). An electrocardiogram showed sinus rhythm. Echocardiography revealed normal cardiac function and no valvular diseases, thrombus, other masses, or congenital diseases. Laboratory studies were almost within normal limits; however, in the dilute Russell viper venom time (dRVVT) assays, which is well known, the lupus anticoagulant test was positive and indicated an APS (Table 1) [2]. The collagen diseases were negative.

**Table 1:** Initial blood laboratory test

	Value	Units	Reference values
Sodium	142	mEq/L	138–145
Potassium	5.3	mEq/L	3.6–4.8
Chloride	105	mEq/L	101–108
Creatinine	0.89	mg/dL	0.65–1.07
Urea	12.9	mg/dL	8.0–20
Glucose	111	mg/dL	73–109
HbA1c	6.4	%	4.9–6.0
Albumin	4	g/dL	4.1–5.1
Total bilirubin	0.7	mg/dL	0.4–1.5
Alanine transaminase	12	U/L	10–42
Aspartate transaminase	17	U/L	13–30
White blood cells	6.7	*10^3/µL	3.3–8.6
Hemoglobin	16.8	g/dL	13.7–16.8
Platelets	419	*10^3/µL	158–348
Prothrombin time	21.4	sec	
PT-INR	1.8		
Activated partial thromboplastin time	38	sec	26–36
Protein S antigen	122	%	73–137
Protein C antigen	100	%	70–150
Protein C activity	101	%	64–146
Dilute Russel viper venom time test	N/A		1.2

We planned to resect the floating mass that caused the embolization at the leg. The safe region to clamp the aorta was marked by the intraoperative transesophageal echocardiogram and epiaortic ultrasonographic scanning. It was not possible to clamp the aorta distal to the thrombus. Cardiopulmonary bypass was established by cannulating the right common femoral artery and right atrium and cooling the body to 20 °C because graft replacement of the ascending aorta was possible without selective cerebral perfusion. Under deep hypothermic circulatory arrest, the aorta was cross-clamped at the level of the pulmonary artery to inject the cardioplegia, and the heart was arrested. After clamping the brachiocephalic artery (BCA) and left common carotid artery, the distal ascending aorta was transected transversely at the BCA level. The mass was found to be attached by a small stalk to the posterior aortic wall at the level of the brachiocephalic trunk. The mass was resected, and there was no macroscopic injury of aortic tunica intima or focal aortic dissection. We closed the aortotomy instead of the graft replacement, and the patient was successfully weaned from the cardiopulmonary bypass.

**Fig. 2:**
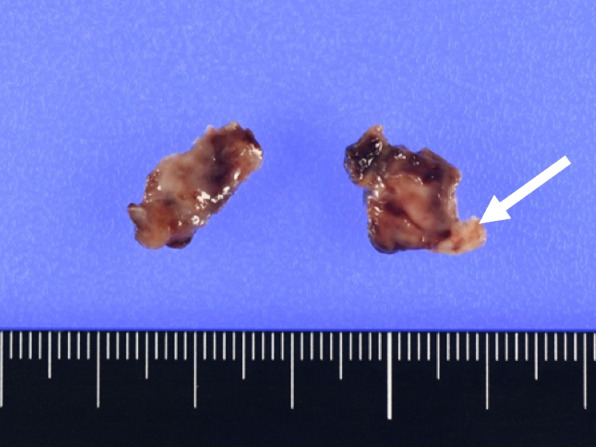
Organizing thrombus (25 × 15 mm) and the aortic attachment site (white arrow)

Histopathological examination revealed that the mass was an organized thrombus (Fig. 2), and the intima of aorta was normal. The claudication of the left lower limb had disappeared after the operation, and the ankle-brachial index was 0.9. He was uneventfully discharged to home on day 10 with aspirin and warfarin therapy because of APS. CTA 1 year after surgery showed no recurrence of thrombus.

## Discussion

The aortic thrombus remains a rare case in the non-presence of an aneurysm, a dissection, or an atherosclerotic aorta. Therefore, there is little consensus on the optimal management of the disease. In a study series of 78 patients, Bowdish et al. [1] reported most acute mural thrombi were seen within the abdominal aorta (64%), followed by the descending thoracic aorta (28%) and the ascending aorta or aortic arch (8%) [3].

The causes of mural thrombi are reported to be associated with hypercoagulable disorder and medication-induced hypercoagulable states such as chemotherapy, estrogen, radiotherapy, and cancer. Bladder cancer and APS are possible causes of the mural thrombi in him. The relationship between cancer and APS is still unclear because of its rarity. Therefore, it was very difficult to determine which factors caused the thrombus. However, postoperative adjuvant chemotherapy was completed 10 years ago for bladder cancer. Given the bladder cancer has been complete remission, we considered APS was the primary cause of mural thrombosis. Most reports of thrombosis in APS are associated with venous or peripheral arterial thrombosis, and there are almost no reports of thrombosis of the aorta [4, 5]. Because of its rarity, there is little consensus on the optimal management of an aortic thrombus. Treatments consist of medical therapy (i.e., anticoagulation therapy) or surgical treatment. It has been reported that anticoagulation alone without thrombectomy may lead to thrombus dissolution in 71.4–100% [1, 6]. Unfortunately, peripheral arterial embolization may still occur and, in some cases, can be fatal. Some articles reported that the recurrent embolic episodes were seen in 25.7–39.0% [7–9] of patients in the anticoagulation group and only in 0–9.1% [7–10] of those in the surgical intervention. However, there is no consensus of the methods of surgical intervention: graft replacement or thrombectomy. According to the principle of Virchow’s triad for thrombogenesis (hypercoagulability, stasis of blood flow, and endothelial injury), the less the endothelial injury, the less is the possibility of recurrence of thrombi. The graft replacement might cause endothelial injury at proximal and distal anastomotic lines. On the other hand, only thrombectomy could cause endothelial damage at the closure line of the aortotomy and the site of thrombus attachment. In this case, if there were a rigid stalk on the aortic lumen, there was the possibility of recurrence of thrombus. We removed the stalk from the aortic lumen and could confirm no injury aortic lumen. Therefore, we did not perform graft replacement to decrease anastomosis lines.

## Conclusions

We experienced a rare case of floating ascending aortic thrombi. As in this case, we consider that a floating ascending aortic thrombus with embolic events should be performed by surgical intervention.

## Data Availability

There are no additional data to disclose.

## Abbreviations

APS: Antiphospholipid syndrome
BCA: Brachiocephalic artery
CTA: Computed tomography angiography
dRVVT: Dilute Russell viper venom time
